# Preschool Hand Function Assessment Instruments: A Scoping Review With Targeted Analysis of Performance‐Based Instruments

**DOI:** 10.1155/oti/4114099

**Published:** 2026-05-07

**Authors:** Marieta Visser, Robyn Smith, Daleen Casteleijn

**Affiliations:** ^1^ Department of Occupational Therapy, School of Health and Rehabilitation Sciences, Faculty of Health Sciences, University of the Free State, Bloemfontein, South Africa, ufs.ac.za; ^2^ School of Health and Rehabilitation Sciences, Faculty of Health Sciences, University of the Free State, Bloemfontein, South Africa, ufs.ac.za; ^3^ Department of Occupational Therapy, School of Healthcare Sciences, Faculty of Health Sciences, University of Pretoria, Pretoria, South Africa, up.ac.za

**Keywords:** fine motor skills, hand function assessment, low- and middle-income countries, performance-based instruments, preschoolers, scoping review

## Abstract

Hand function is a critical determinant of preschool children′s participation in everyday occupations. Accurate hand function assessment is vital for guiding interventions, yet assessment practices are highly variable. Despite the abundance of instruments, only a few standardised performance‐based tools assess all hand function components. No comprehensive mapping exists of key preschool instrument specifications to guide clinicians and researchers, particularly those working in diverse contexts. The study mapped all published hand function–relevant instruments used with children aged 3–5 years, including pathology‐specific measures, proxy‐report tools, classification systems, and multidomain developmental assessments. Employing scoping review methodology, 13 databases were searched, and all retrieved sources up to September 2024 were managed on Covidence. Two independent reviewers conducted title, abstract and full‐text screenings based on eligibility criteria. The search found 810 sources, with 84 meeting inclusion criteria, identifying 62 instruments. Most were performance‐based (*n* = 46; 74.2%), followed by proxy measures (*n* = 11), classifications (*n* = 3) and two unspecified. Over a third targeted children with health conditions, whilst 22 covered both typical and atypical development. Sources from the Global South and LMICs were notably scarce. Performance‐based measures were examined in greater depth, with only one addressing all hand function domains. Findings highlight the need for a standardised, culturally adaptable performance‐based tool that reflects preschool children′s real‐world occupations and is applicable across diverse settings. This review offers a structured foundation for developing or adapting such a tool, bridging current gaps in assessment practice in both high‐ and low‐resource environments.

## 1. Introduction

Achieving inclusive and equitable quality education for all begins with establishing strong foundations in the earliest years of a child′s life, ensuring that every child acquires the knowledge and skills necessary for lifelong learning [1]. Foundational skills encompass basic abilities such as cognitive, language, socioemotional and motor skills, all developing in early childhood, forming the groundwork for success in school and throughout life [2]. The current global emphasis on improving early childhood education and development (ECED) outcomes has also translated to the occupational therapy profession, with increasing priority being placed on assessing preschoolers′ (3–5‐year‐old children) development and academic performance, including fine motor skills [2, 3].

Fine motor skills, particularly hand function skills, are vital for performing daily life activities. Hand function evolves across the lifespan to meet the demands imposed by changing social and technological environments. The term “hand function” is used interchangeably with hand skills, dexterity and fine motor skills/coordination. Hand function is defined as the voluntary movements of the hands to manipulate objects during a specific task and/or nonobject‐related movements where the hands are used for body contact or in interaction with the environment and persons [4–6].

Hand function is a broad term encompassing many components, including physical structures, biomechanics, muscle tone and strength, sensation, range of motion, hand arches and grips and grasps. It also includes more complex skills such as voluntary release, in‐hand manipulation (IHM) and bimanual coordination. Adequate hand function depends on postural control, as well as visual–perceptual and cognitive development [7]. Chien et al. [3] distinguish between nonobject‐related hand function, such as manual gestures and body‐contact skills, and object‐related hand function, which includes arm–hand use (e.g., throwing), hand skills (e.g., grasping), bimanual coordination and overall quality of hand function, such as speed and coordination. In contrast, Kimmerle et al. [5] emphasise the importance of task parameters, including task demands, purpose, type of movements (e.g., repetitive actions or sequences of different movements) and the object or tool used. Tsang [6] further highlights the significance of the child′s developmental level and maturation when assessing hand function.

The preschool years are a period characterised by rapid maturation of several body functions and the development and mastery of a number of skills, including hand function, enabling the child to participate in preschool‐related tasks [5, 8, 9]. Preschool children spend more than a third of their time engaging in fine motor activities such as self‐care, manipulating objects and paper‐based activities and media usage (i.e., smartphone, computer and tablet), requiring well‐developed hand skills [7, 10, 11]. This need becomes more pronounced in later school years as time spent on written work (and media‐related screen time) increases, demanding more speed, quality and endurance [7]. Moreover, research has consistently emphasised the value of fine motor skills in predicting preschool‐aged children′s performance in activities essential for school readiness (e.g., writing, cutting and tying shoelaces) and self‐help skills (eating, dressing and toileting) [3, 12].

However, a child′s hand function can be negatively affected by factors associated with poverty, developmental delays, disability or disease. It is estimated that nearly two‐thirds of children in sub‐Saharan Africa are at risk of not reaching their full developmental potential due to the intersecting effects of social determinants of health (SDOH) and inadequate nurturing care. The SDOH, including poverty, undernutrition and unsafe environments, create structural barriers that shape health and developmental outcomes, whilst nurturing care encompasses the immediate provision of responsive caregiving, adequate nutrition, safety and opportunities for early learning [13].

In South Africa, inequitable, inadequate and inaccessible early childhood education (ECE) exacerbates the risk of children falling short of their developmental potential [14]. Reports confirmed suboptimal development across cognitive, motor (gross and fine) and socioemotional domains amongst many South African preschool children, likely compromising their preparedness for formal schooling, limiting future scholastic performance and their trajectory towards healthy and productive adulthood [14–16]. Recent data from the “Thrive by Five” index for South Africa highlighted that only 30.4% of South African preschoolers′ fine motor skills were on par for age [14].

Occupational therapists are skilled at discerning subtle hand movements during task performance, allowing them to evaluate hand function in children [6, 11]. They often assess and treat preschool‐aged children experiencing limitations in activities requiring fine motor skills such as writing, cutting or fastening buttons [4, 5]. Accurate hand function assessment is critical to the planning of appropriate contextualised intervention tailored to address specific identified physical impairments, activity limitations and participation restrictions, thereby promoting overall occupational engagement in early childhood [9]. Moreover, regular reassessment throughout the intervention continuum has proven necessary in gauging the efficacy of hand function interventions in improving children′s daily occupations [17]. However, little is known globally about how occupational therapists select and use assessment tools to evaluate preschool children′s hand function and to monitor intervention outcomes, thereby determining the efficacy of therapeutic approaches [18].

Evaluation and monitoring of early childhood development and educational performance, both at the individual level (e.g., in clinics) and the population level (e.g., across preschool programmes), are essential for achieving global, regional and national goals aimed at improving early outcomes. Furthermore, preschool developmental screening and more detailed evaluations, where indicated, are widely advocated as essential steps in the early identification of developmental delays and functional limitations [2, 14, 16]. Such early detection enables timely, targeted interventions that can improve children′s participation in key occupations. Although many developmental assessment instruments and hand function assessment tools are available for preschool‐aged children, it remains unclear whether they provide a truly comprehensive evaluation of hand function that also accounts for linguistic and cultural nuances.

Against the backdrop, occupational therapists in low‐ to middle‐income countries (LMICs) (e.g., South Africa) need a comprehensive, standardised, affordable, culturally and contextually adaptable performance‐based assessment instrument to evaluate preschool children′s hand function [19]. Performance‐based measures are essential in occupational therapy because they provide objective, real‐time insight into a client′s functional abilities rather than merely what they report they can do, guiding personalised and effective intervention planning.

In light of this need, a scoping review was considered the most appropriate evidence synthesis method because of the breadth and conceptual complexity of the topic to systematically identify and map available assessment tools and their key specifications. Given the exploratory nature of the review and the anticipated heterogeneity of the tools, study designs and contexts, a scoping review was deemed most appropriate in line with methodological guidance provided by Peters et al. [20] and Tricco et al. [21].

A scoping review would identify whether such instruments exist and, if so, whether current instruments would require adaptation or whether, in the absence of existing instruments, the development of a new instrument is warranted. Although previous reviews have examined hand function assessments for infants, specific pathologies, IHM and occupation‐based evaluations, none have systematically mapped instruments designed specifically for use in preschool‐aged children [22–25]. This scoping review was aimed at mapping the key specifications of all published hand function assessment instruments for preschool‐aged children. In addition, this review conducted a focused mapping and analysis of performance‐based instruments. This analysis identified the specific hand function domains assessed and the corresponding assessment items used. The emphasis on performance‐based measures reflects their clinical value in occupational therapy, where direct observation of task performance provides critical insights for intervention planning. This in‐depth mapping was also necessary because the evidence will be used as the empirical basis for a larger study aimed at adapting or developing a performance‐based hand function assessment tool to measure the occupations and occupational hand performance of preschoolers.

## 2. Methods

A scoping review serves as a knowledge synthesis tool to identify and map all available evidence on a topic [20, 21]. It is also valuable to clarify key concepts, identify key characteristics related to a concept and identify/analyse gaps in existing knowledge [26, 27]. This approach allows exploration of broader research questions using varied methodologies and data qualities [20, 21]. The current review was conducted in accordance with the Joanna Briggs Institute (JBI) methodology for scoping reviews [20] and reported using the Preferred Reporting Items for Systematic Reviews and Meta‐Analyses extension for Scoping Reviews (PRISMA‐ScR) checklist [21].

### 2.1. Review Questions

The research questions for this review were as follows:

• What published assessment instruments are used to assess hand function in preschool‐aged children, and what are their key specifications (including target population, age range, purpose, type of instrument, country of origin and authors)?
• Additionally, which hand function domains and specific assessment items (e.g., movements, tasks and activities) are included in the performance‐based instruments identified?

### 2.2. Information Sources and Eligibility Criteria

The review considered all available published evidence on hand assessment instruments suitable for use in preschool‐aged children, irrespective of gender, ethnicity and health status (typically and atypically developing or children with pathologies). The eligibility criteria were structured using the population–concept–context (PCC) framework.

Population (preschool‐aged children): To be included, the instrument′s target group had to include children aged 3–5 years of age. However, a slightly broader age band, 2–6 years, was included, provided 3–5‐year‐olds fall squarely in the normative sample. Any clinical assessment instrument was eligible, including standardised and nonstandardised tools, outcome measures, proxy‐reported measures and observational checklists.

Concept (hand function assessment instruments): Both outdated and existing instruments for health conditions and disabilities were included to comprehensively map all available assessment tools. Although some assessment tools are no longer used clinically, older instruments were still reviewed because their item content could still inform the development of a comprehensive item pool. By cataloguing both legacy and current tools, domains and individual items could be compared against existing frameworks. This approach ensures that no relevant content is overlooked when adapting existing measures or creating a new instrument.

Context (clinical, research or educational use): The review considered publications from peer‐reviewed journals using all types of study designs and methodologies describing the specific hand function assessment instrument and/or the development and psychometric testing thereof. Many assessment tools are developed by organisations and clinicians and may not be published in peer‐reviewed journals. Grey literature was therefore essential to ensure comprehensive mapping of available tools and to reduce publication bias. This approach is consistent with scoping review methodology, which emphasises broad evidence capture. Grey literature searches included systematic searches of organisational and professional body websites, conference proceedings (e.g., published on the ResearchGate platform or Wiley publishers) and academic repositories of theses and dissertations (e.g., Figshare and ProQuest). Targeted Google searches were performed using predefined search terms. All searches used the same terms as the database search and were screened against the study′s eligibility criteria. The review also considered reviews, theoretical publications and citations from reference lists of identified sources to ensure comprehensive coverage of available instruments, including those that may not have been indexed, are not yet published, or are primarily used clinically rather than through publishing houses or scientific journals.

An upper date limiter was set for publication up to September 2024. To comprehensively map all available assessment tools, the initial database search was conducted without any lower date or language limiters to ensure inclusion of older instruments that had not been updated or non‐English instruments and to minimise the risk of language and publication bias. Where necessary, language restrictions were applied during the screening stage due to feasibility considerations (i.e., only include studies that had been translated). When potentially eligible sources were identified in a language other than English or the full text was not available, efforts were made to obtain an English version, including conducting an additional search or contacting the corresponding author. If no English version could be obtained, the source was excluded from further review. The specific characteristics of the sources of evidence and the type of information sources were evaluated against the study′s eligibility criteria as outlined in Table 1.

**Table 1:** Selection criteria to identify the hand function assessment instruments for preschool‐aged children.

Properties	Inclusion criteria	Exclusion criteria
Population the instrument is intended for		
Age range included in the target population of the instrument	Children aged 2–6 years	• Infants (0–23 months) • Children older than 6 years • Adults
Country/ethnic group	All	None
Setting	All (geographical context, health system or healthcare setting)	None
Gender	All	None
Children with any medical conditions and/or disabilities	All	None
Type and purpose of the instruments		
	• Hand skills/function assessment instruments, including standardised, nonstandardised, performance‐based, clinical assessment instruments, outcome measures, proxy‐reported measures and observational checklists • Global developmental assessment instruments that assess multiple domains of a child′s development	• Instruments that only measure body function, that is, the dynamometer, range of motion and manual muscle testing • Assessment instrument for upper extremity function, that is, shoulder/elbow • Handwriting, handwriting speed or dysgraphia assessment instruments • Hand preference instruments • Assessment protocols/observations only used for a specific research study
Type of article/source		
Publication date	All years up to March 2024	Publications after March 2024
Publication type	• Publications from peer‐reviewed journals • Grey literature (unpublished theses/dissertations, conference proceedings and web‐based sources) • Publications from reference lists	
Text	All sources with full text available	Full text not available
Language	All languages	English translated version unavailable
Study design	All types of study designs and methodologies	Studies on the translation of the original assessment instrument
	Reviews and theoretical publications	Hand function assessment models/frameworks for children

Sources were excluded if they reported on (i) frameworks or models of hand function; (ii) hand assessment instruments only for children younger than 2 years, older than 6 years and adults; (iii) instruments aimed to specifically assess body function/structures (e.g., muscle strength) or upper extremity function (not including hand function), hand preference and handwriting and (iv) instruments that were translated from the original instrument but similar in structural information to avoid duplication of instruments and overestimation of the number of available tools (e.g., the Italian version of the ABILHANDS‐kids).

**Figure 1:**
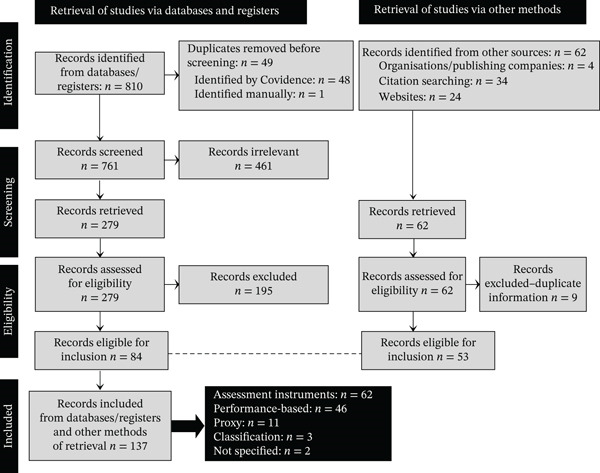
Preferred Reporting Items for Systematic Reviews and Meta‐Analyses extension for Scoping Reviews (PRISMA‐ScR) flow diagram.

For this review, *sources* refer to the broad range of sources of evidence meeting the eligibility criteria and are not limited to empirical research studies. *Records* (as presented in Figure 1, PRISMA flow diagram) refer to the individual entries retrieved from databases, registers, websites or other information sources. *Studies or articles* refer to the full‐text publications that were included, charted, analysed and reported in the results.

### 2.3. Search Strategy

With the assistance of an experienced medical information specialist, a comprehensive search strategy was developed and implemented to locate all existing published sources on the topic of interest. Searches were conducted in multiple bibliographic databases via the EBSCOHost platform, including Academic Search Ultimate, Africa‐Wide Information, APA PsycArticles, APA PsycInfo, APA PsycTests, Applied Science & Technology Source Ultimate, CINAHL with Full Text, ERIC, Health Source: Nursing/Academic Edition, Humanities Source Ultimate, MEDLINE, Sociology Source Ultimate and SPORTDiscus with Full Text. It is acknowledged that it is not feasible to search all possible information sources; therefore, key databases relevant to the review topic were selected. Reference lists of included sources were also screened to identify additional relevant literature.

The following search string was used:

ti (child∗ or paediatr∗ or pediatr∗) (scale or scales or tool∗ or instrument∗ or test or tests or inventor∗ or questionnaire∗) ((“hand function∗” or “GRIP∗ strength∗” or “pinch∗ strength∗” or “range of motion” or “Hand∗ Strength∗” or “Power grip” or “hand skill∗” or dexter∗ or “hand anthropometr∗” or “grasp∗ force” or “grasp∗ strength∗” or “bimanual perform∗” or “hand perform∗” or “hand capacity” or “fine motor∗” or “hand integrity” or “integrity of hand” or “in‐hand manipulat∗” or “inhand manipulat∗” or “in hand manipulat∗” or bilateral∗) n4 (evaluat∗ or assess∗ or measur∗ or screen∗)) (hand or hands or manual) and (preschool∗ or pre‐school∗ or “pre school∗”).

An initial search was followed by an analysis of specific text words contained in both the titles and abstracts of relevant sources and the index terms used to describe the sources. The preliminary search identified 615 potential sources for possible inclusion (after automated system duplicates were removed), suggesting good feasibility and relevance of the search strategy. The final search employing similar databases and search strings was conducted in collaboration with an information librarian in March 2024 and identified 810 potential sources.

### 2.4. Selection of Sources of Evidence

All identified records were imported into the Covidence (Melbourne, Australia) web‐based software data management platform, and duplicates were removed by the primary reviewer. A first pilot test was conducted by two reviewers, who screened four titles and abstracts against the eligibility criteria. Following the pilot test, the two reviewers independently screened the titles and abstracts of retrieved records against the eligibility criteria.

A second pilot was done by the two reviewers, who screened four full‐text sources against the eligibility criteria. Minor changes were made to the criteria to accommodate unexpected characteristics of the sources (e.g., full‐text access restricted by an embargo). All potentially relevant full‐text sources were then assessed. The 35 discrepancies between reviewers regarding eligibility were minor and were resolved through regular discussion between the two reviewers, both experts in the field. Consensus on all sources was achieved without requiring an independent third reviewer. To enhance transparency, clarity and consistency in reporting, the review followed the PRISMA‐ScR reporting guidelines (Figure 1).

### 2.5. Data Extraction and Charting

The data extraction tool was developed by the primary reviewer on Covidence. The tool was divided into subsections, each with relevant identified data points relating to the review objectives. The first subsection included general information about the extracted sources (i.e., authors, study title, year, country of research and the Covidence number). The second subsection included the key specifications of the assessment instrument (i.e., name of the instrument, abbreviation of the instrument, edition/version of the instrument, age parameters, target population, purpose of the instrument, type of instrument, country of origin and the primary author/s of the instrument). The third subsection pertained to the domain of hand function assessed and the items used in the instrument.

A third pilot was conducted by both reviewers on Covidence using the first six sources, selected according to the alphabetical order of authors, to ensure that all relevant data items were captured and that the charting process was applied consistently between reviewers. Following the pilot exercise, the form was refined before being applied to the remaining sources of evidence. Subsection 1 performed well, whilst Subsection 2 had to be adapted from allowing six instruments to allowing 10 instruments captured per article, since some articles reported on multiple instruments. However, three articles (one systematic and two scoping reviews) reporting on more than 10 instruments were captured by hand. No disagreements occurred between the reviewers to be resolved through discussion or by a third reviewer. Following the pilot, both reviewers screened all full‐text articles for eligibility.

Data extraction was conducted primarily by one reviewer, with a second reviewer verifying 10% of the full‐text extractions to ensure accuracy. Data were captured between April and December 2024 and downloaded from Covidence on a password‐protected CSV (comma‐separated values) Excel spreadsheet (Microsoft Corporation; Redmond, WA, United States). The exported data were cleaned for duplications, and the included instruments′ key specifications were organised in Excel documents according to the instruments′ names sorted alphabetically, abbreviation, edition/version, age parameters, target population, purpose of the instrument, type of instrument, country of origin and their primary author/s.

During data charting, some included sources provided limited information on the characteristics of the measurement instruments. To address these gaps, additional information was obtained from publicly available resources such as instrument manuals, publisher websites, and materials provided by relevant organisations, as outlined in Figure 1. These sources were used only to verify or supplement instrument‐related information and were not included as independent sources of evidence in the review.

### 2.6. Critical Appraisal of Sources of Evidence

Descriptive information about the included sources, including study design, sample size and research context, was extracted to provide an overview of the characteristics of the evidence base. Consistent with the objectives of a scoping review and guidance from the JBI, no formal critical appraisal of the methodological quality of the included sources was conducted.

### 2.7. Data Analysis and Presentation

In this review, the primary unit of analysis was the hand function assessment instrument rather than the individual source or multiple sources reporting on the same instrument. The number of included sources is reported for context to indicate the extent of the evidence available for each instrument. The PRISMA‐ScR flow diagram (Figure 1) was compiled to report the identification, screening and selection process of the sources (also referred to as records in Figure 1) and the assessment instruments at each stage of the review [28].

The charted data were analysed using descriptive numerical summaries and thematic categorisation to map the characteristics and key concepts of the included sources. Extracted information was organised according to key variables such as publication year, geographical setting, study design and main findings. Frequency counts were used to summarise the distribution of sources across these categories. The findings are presented using tables and narrative summaries to provide an overview of the available evidence and highlight emerging themes relevant to the review objectives.

## 3. Results

A PRISMA‐ScR flow diagram was used to illustrate the search and selection process, including the number of records identified, screened, assessed for eligibility and included in the review, along with reasons for exclusion at each stage [21] (Figure 1). The database search yielded 810 records that were imported to Covidence, of which 49 duplicates were removed, leaving 761 titles and abstracts to screen. After screening, 461 were deemed irrelevant, and 300 full‐text records were assessed for eligibility, of which 195 were excluded. A further 62 records were identified through other sources, of which nine were excluded after full‐text review. In total, 204 records were excluded for the following reasons (in this section of the results, the unit of analysis refers to the records—i.e., the sources of evidence included in the review—and not to the assessment instruments identified in the record):

• Full text was not available, or not available in English, or access‐restricted by an embargo (*n* = 21).
• The age category of the study population was 6 years and above or for adults (*n* = 16).
• The assessment used in the study was not hand function–specific or only assessed a body function–related aspect such as muscle strength (*n* = 11).
• The instrument/observation was still under development or was only developed for the specific study being reported (*n* = 9).
• Informal hand function observations without a clear indication of an instrument (*n* = 6).
• No indication of how hand function was assessed and details about the instrument (*n* = 6).
• Translated version of an existing instrument or a translation study of an existing instrument where information was not available on the original instrument (*n* = 6).
• A hand function assessment framework or model and not an instrument (*n* = 2).
• The instrument referred to in the record has already been captured (*n* = 127). For this study, the first article mentioning an instrument, based on Covidence′s alphabetical order, was included. (Potential additional information about the instrument in subsequent publications was stored on Covidence.)

### 3.1. Overview of Included Sources

A total of 137 sources were included in the review (see Figure 1). Sources included from the database search (*N* = 84) were published between 1976 and March 2024, with a substantial proportion (34, 40.5%) published during 2010–2019, followed by 23 (27.4%) between 2020 and 2024. The scoping review allowed for multiple methodologies to be included, with most sources (*n* = 28; 33.3%) reporting on intervention designs, followed by descriptive designs (*n* = 25; 29.8%), and psychometric testing and instrument descriptions (*n* = 23; 27.4%), as summarised in Table 2.

**Table 2:** Characteristics of database sources included (*N* = 84).

**Publication year**	**n (%)**	**Country of study^a^**	**n (%)**
1976–1979	1 (1.2)	United States	18 (20.6)
1980–1989	0 (0)	Turkey	9 (10.3)
1990–1999	10 (11.9)	Australia	8 (9.2)
2000–2009	16 (19.0)	United Kingdom	5 (5.7)
2010‐2019	34 (40.5)	Canada	5 (5.7)
2020–2024	23 (27.4)	Brazil	4 (4.6)
		Sweden	4 (4.6)
		Saudi Arabia, South Africa and Egypt	3 (3.4) each
		The Netherlands, Norway, Taiwan, Belgium and France	2 (2.3) each
		Zimbabwe, South Korea, Finland, Italy, China, Japan, Malaysia, Germany, Norway, India and Poland	1 (1.1) each
		Not stated	4 (4.6)
**Type of studies**			**n (%)**
Descriptive studies on children with or without medical conditions or disabilities focusing on their motor development, performance and the factors influencing their development.			25 (29.8)
Intervention studies, such as the outcome of hand function after surgery or therapy.			28 (33.3)
Descriptive studies on hand function assessment practices and comparisons of children′s hand function using different instruments.			4 (4.8)
Psychometric testing of instruments or descriptive studies about an instrument.			23 (27.4)
Reviews studies (systematic and scoping reviews).			4 (4.8)

^a^The total number of articles according to country was 87 because some studies were conducted in more than one country.

The sources were conducted across several countries, as shown in Table 2. Sources were also categorised according to Global North versus Global South countries [29, 30] and high‐income versus low‐/middle‐income status [31] to explore whether the origins of assessment tools align with the economic resources and broader sociocultural contexts in which they are intended to be used. Whilst physiological aspects of hand function do not differ by geography, contextual and cultural practices, including typical childhood occupations, available materials and service delivery models, may influence the design, content and applicability of assessment instruments. It was found that the majority of sources originated from the Global North (*n* = 55; 67.1%) compared to the Global South (*n* = 27; 32.9%) and from high‐income countries (HICs) (*n* = 59; 71.9%) versus LMICs (*n* = 23; 28.1%). As reflected by the summary presented in Table 3, these findings highlight a clear under‐representation of sources from the Global South and of tools used in LMIC contexts.

**Table 3:** Distribution of sources (*N* = 82) according to Global North and Global South [29, 30]) and high‐income and low‐/middle‐income [31] countries.

**United Nations classification**
Global North (*n* = 55; 67.1%)
United States (*n* = 18)
Australia (*n* = 8)
Canada and the United Kingdom (*n* = 5 each)
Sweden (*n* = 4)
The Netherlands, Norway, Belgium, France and Taiwan (*n* = 2 each)
Finland, Italy, Germany, Japan, South Korea, and Poland (*n* = 1 each)
Global South (*n* = 27; 32.9%)
Turkey (*n* = 9)
Brazil (*n* = 4)
Saudi Arabia, South Africa and Egypt (*n* = 3 each)
China, Malaysia, India and Zimbabwe (*n* = 1 each)
**World Bank classification**
High‐income countries (*n* = 59; 71.9%)
United States (*n* = 18)
Australia (*n* = 8)
Canada and the United Kingdom (*n* = 5 each)
Sweden (*n* = 4)
Saudi Arabia (*n* = 3)
The Netherlands, Norway, Belgium, France and Taiwan (*n* = 2 each)
Finland, Italy, Germany, Japan, South Korea and Poland (*n* = 1 each)
Low‐/middle‐income countries (*n* = 23; 28.1%)
Upper‐middle income
Turkey (*n* = 9)
Brazil (*n* = 4)
South Africa (*n* = 3)
China and Malaysia (*n* = 1 each)
Lower‐middle income
Egypt (*n* = 3)
India (*n* = 1)
Low‐income
Zimbabwe (*n* = 1)

### 3.2. Identification and Characteristics of Assessment Instruments From Included Sources

From the 84 database‐identified sources, 76 hand function assessment instruments were identified that met the eligibility criteria. Some sources reported the use of multiple instruments, whilst several instruments were used across several sources. However, whilst supplementing the data extracted from the database‐identified sources with information obtained from secondary sources (such as related publications, technical manuals or publicly available documentation) of the same instrument (Figure 1), it became evident that some of these 76 instruments were either designed for children older than 6 years, intended for adults or not specifically focused on hand function as initially indicated. Hence, 15 instruments were subsequently excluded. Furthermore, one additional instrument not identified in the data‐based sources was identified from a reference list, confirmed via a website and included, resulting in a total of 62 included assessments (Table 4).

**Table 4:** Key specifications of the included assessment instruments (*N* = 62).

No.	Name of instrument	Abbr.	Edition/version	Age group	Target population	Purpose of the instrument	Country	Primary author/s
*Performance-based instruments (* *n* = 46 *)*								
1	Annett Pegboard or Peg Moving Task	PEGS, PMT and PMT‐5	10‐holes x2 version 5‐holes x2 version	3–24 years	NS	To assess bilateral hand function, hand preference and hand skill asymmetry	United Kingdom	[32]
2	(Annett PMT) Computerised Peg Moving Task	CPMT	Adapted version of PMT	NS	NS	To assess bilateral hand skills	NS	[33]
3	Assessment of Children′s Hand Skills	ACHS	First ed.	2–12 years	Children with and without disabilities	To assess children′s hand use when engaged in daily activities in natural contexts	Australia	[34]
4	Assisting Hand Assessment	AHA	Version 5.0	18 months to 12 years	Children with hemiplegic or unilateral CP and obstetric BPP	To assess how effectively a child makes use of their affected hand during bimanual performance	Sweden	[35]
5	Bayley Scales of Infant and Toddler Development	Bayley	First ed. reported Fourth ed. latest	1–42 months	Infants and toddlers with or without developmental delay	To assess and identify developmental delays	United States	[36]
6	Besta Scale	NS	NS	18 months to 12 years	Children with hemiplegic CP	To assess the hand function of the affected and unaffected hands during activities of daily living	Italy	[37]
7	Bruininks‐Oseretsky Test of Motor Proficiency	BOTMP	BOTMP and BOT‐2 reported BOT‐3 latest	4 and 21 years	Children with and without motor problems	To assess a broad array of fine and gross motor skills	United States	[38]
8	Both Hands Assessment	BoHA	NS	18 months to 12 years	Children with bilateral CP	To assess how children with bilateral CP use their hands in bimanual activities and the difference between hands	Norway	[39]
9	Box and Block Test	BBT	NS	6 to adults	Children with or without a neurological diagnosis	To assess unilateral manual speed and dexterity	NS	[40]
10	Brachial Plexus Outcome Measure	BPOM	Version 2.0	4–19 years	Patients with obstetric BPP	To assess the functional activity of the shoulder, elbow, forearm, wrist and hand	Canada	[41]
11	Brazilian Scale for Motor Development (Escala de Desenvolvimento Motor)	MDS/EDM	First ed.	2–11 years	Children with typical and atypical motor development	To assess the domains of psychomotricity: Fine motor, overall motor, balance, body scheme, spatial organisation, temporal organisation and laterality	Brazil	[42]
12	Computer game–based upper extremity assessment of manual dexterity	CUE	NS	NS	Children with CP	To assess manual dexterity	NS	[43]
13	Developmental Hand Function Test	DHFT	NS	NS	NS	To assess functional hand movements	NS	NS
14	Early Childhood Developmental Criteria	ECDC	First ed.	3–5 years	Environmentally disadvantaged children	To assess cognitive and motor development	South Africa	[44]
15	Erhardt Developmental Prehension Assessment	EDPA	First and second ed.	Birth to 6 years	Children with CP or other motor disorders	To measure components of arm and hand development	United States	[45]
16	Functional Dexterity Test	FDT	NS	3–17 years and adults	NS	To assess dexterity	NS	[46]
17	Grooved Pegboard	NS	NS	5 years to adulthood	NS	To assess dexterity	NS	[47]
18	In‐hand Manipulation Test Quality section	IMT‐Q	NS	3–8 years	NS	To assess in‐hand manipulation skills	United States	[48]
19	Jebsen‐Taylor Hand Function Test	JTHFT	Adapted ed. for children	6–65+ years	Adults and children with and without motor impairments	To assess broad aspects of hand function commonly used in everyday activities	NS	[49]
20	Kobe Hand Function Test	NS	NS	4–90 years	Children and adults with or without hand dysfunction	To assess the time required to do hand function tasks of daily living	Japan	[50]
21	Malawi Developmental Assessment Tool	MDAT	NS	0–6 years	Children with or without a neurological diagnosis	To assess child development in four domains: Fine and gross motor, language and social	Malawi	[51]
22	Melbourne Assessment of Unilateral Upper Limb Function	MAUUF	First and second ed.	5–15 years	Children with CP or other neurological impairments	To assess the quality of unilateral upper limb movements	NS	[52]
23	Modified In‐Hand Manipulation Test	TIHM‐M	NS	6–9 years	Children without or with a neurological diagnosis	To assess in‐hand manipulation	Switzerland	[53]
24	Motor Performance Checklist	MPC	NS	4–11 years	Typical developing children and those at risk for DCD	To assess gross and fine motor skills to identify children at risk of DCD	Australia	[54]
25	Movement Assessment Battery for Children	MABC, MABC‐2 and MABC‐3	NS Second ed. Third ed.	4–12 years 3–16 years 3–25 years	Children with potential motor skill deviations	To assess motor skills and coordination to identify motor disorders in children	United Kingdom	[55] [56]
26	Nine‐Hole Peg Test	NHPT	NS	5–65 years	Children and adults with or without disabilities	To assess dexterity and hand–eye coordination as a measure of hand function	United States	[57]
27	Observation Protocol: In‐Hand Manipulation and Functional Skill Development	NS	NS	2–7 years	NS	To assess in‐hand manipulation	United States	[58]
28	Peabody Developmental Motor Scales	PDMS, PDMS‐2 and PDMS‐3	First ed. Second ed. Third ed.	0–83 months 0–71 months 0–5 years	Typically developing children	To assess motor abilities that develop early in life to identify children with delayed motor development	United States	[59, 60]
29	Paediatric Arm Functional Test	PAFT	First ed.	2–6 years	Children with CP	To assess the capacity to complete actions and tasks with the more‐affected arm	United States	[61]
30	PizzaPutty Test	NS	NS	Children of all ages	Children with features of upper limb involvement	To assess hand function grip	NS	[62]
31	Purdue Pegboard	PPT	NS	5–89 years	People with or without a variety of conditions (physical/psychological)	To assess manual dexterity	United States	[63]
32	Quality of Upper Extremity Function	QUEST	NS	18 months to 8 years	Children with spasticity due to neuromotor dysfunction such as CP	To assess movement patterns and hand function	Canada	[64]
33	Shriners Hospital for Children Upper Extremity Evaluation	SHUEE	NS	3–18 years	Children with hemiplegic CP	To assess pre‐ and postintervention of spontaneous functional (SFA), dynamic positional (DPA) and grasp and release analysis (GRA) of the affected upper extremity	United States	[65]
34	Strength–Dexterity Test	SD test	NS	4–17 years and adults	Typically developing children (for the study)	To assess dexterity (dynamic force control and precision)	Sweden	[66]
35	Task‐Based Bilateral Fine Motor Skill Assessment	TBA	NS	5–6 years	Typically developing South African children	To assess the school readiness of fine motor skills in school‐based tasks	South Africa	[67]
36	Test of In‐Hand Manipulation	TIHM	NS	4–6 years	NS	To assess in‐hand manipulation	United States	[68]
37	Timed‐Test of In‐Hand Manipulation	T‐TIHM	NS	5–6 years	Typically developing children	To assess in‐hand manipulation	The Netherlands	[69]
38	Test of In‐Hand Manipulation—Revised	TIHM‐R	NS	3–7 years	NS	To assess in‐hand manipulation	Australia	[70]
39	Test of In‐Hand Manipulation Skills	TIMS	NS	3–9 years	Typically and atypically developing children	To assess in‐hand manipulation	India	[71]
40	Test for Manual Dexterity in Visually Impaired Children	NS	First ed.	6–12 years	Children with visual impairments	To assess manual dexterity	The Netherlands	[72]
41	The Bead Maze Hand Function Test for Children	BMHF	First ed.	4–15 years	Typically developing children	To assess movement quality and control of forces applied whilst moving a bead over wires	United States	[73, 73]
4,2	Toddler and Infant Motor Evaluation	TIME	First ed.	4–42 months	Children with developmental delays	To assess gross and fine motor skills	United States	[74]
43	Timed Test of In‐Hand Manipulation	TIHM	NS	5–6 years	NS	To assess in‐hand manipulation skills	NS	[75]
44	Unilateral Below Elbow Test	UBET	NS	NS	Children with unilateral congenital BED	To assess arm or prosthetic function in children with unilateral limb deficiencies	NS	[76]
45	University of the Free State In‐Hand Manipulation Checklist	UFS IHM‐C	First ed.	4–7 years	NS	To assess in‐hand manipulation	South Africa	[77]
46	Unnamed in‐hand manipulation test of Pehoski et al.	NS	NS	3–6 years	NS	To assess in‐hand manipulation	United States	[78, 79]
*Proxy measures (* *n* = 11 *)*								
47	ABILHAND‐Kids	NS	First ed.	6–15 years	Children with CP with upper limb impairments	To assess manual ability as perceived by the parents	NS	[80]
48	Ages and Stages Questionnaire	ASQ	Third ed.	1 month to 5.5 years	NS	To screen the development of children using parents′ input	United States	[81]
49	Ankara Developmental Screening Inventory	AGTE	NS	0–6 years	NS	To assess children′s development and possible developmental problems	Turkey	[82]
50	Caregiver Functional Use Survey	CFUS	NS	NS	Children with hemiplegic CP	Parental perceptions of how much and well their child used the more affected upper extremity in bimanual and unimanual tasks	NS	[83]
51	Childhood Health Assessment Questionnaire Parent–caregiver version PV Patient–child version CV	CHAQ	CHAQ PV CHAQ CV	1–12 years 12+ years	Children with chronic diseases such as juvenile idiopathic arthritis	To assess functional ability and quality of life. It measures the impact of a child′s health condition on their daily activities and overall well‐being	Italy	[84]
52	Children′s Hand Skills Ability Questionnaire	CHSQ	First ed.	2–12 years	Children presenting with a range of disabilities	To assess children′s manual ability when engaging in activities within a real‐life context	Australia	[85]
53	Children′s Hand‐Use Experience Questionnaire	CHEQ	CHEQ CHEQ 2.0 Mini CHEQ	6–18 years 6–18 years 3–6 years	Children and adolescents with unilateral hand dysfunction	To assess children and adolescents′ perceived experience of performing typical bimanual activities	NS	[86]
54	Hand Use at Home Questionnaire	HUH	NS	3–10 years	Children with neonatal BPP or unilateral CP	To assess the amount of spontaneous use of the affected hand in activities	The Netherlands	[87]
55	Parent Evaluation of Developmental Status	PEDS	PEDS revised	Birth to 8 years	NS	To assess parental concerns about the child′s development	United States	[88]
56	Patient‐Reported Outcomes Measurement Information System: Paediatric Upper Extremity Questionnaire	PROMIS	NS	5–17 years	Children living with chronic conditions or those typically developing	To assess children′s upper limb function during daily activities	United States	[89]
57	Paediatric Upper Extremity Motor Activity Log	PMAL	First ed. revised ed.	7 months to 8 years	Children with hemiplegic CP	Parental perceptions of how much and well their child uses the more affected upper extremity in unimanual tasks	United States	[90]
*Classification (* *n* = 3 *)*								
58	Bimanual Fine Motor Function	BFMF	Version 2	3–18 years	Children with CP	Classification of hand function in children with CP into five different levels	Norway	[39]
59	Manual Ability Classification System	MACS	First ed.	4–18 years	Children with CP	Classifies hand function in different severity levels based on handling objects in daily activities	Sweden	[91]
60	Raimondi hand function scale	NS	NS	0–24 months	Conditions such as neonatal brachial plexus palsy	To classify hand function deficits on a scale from complete paralysis to active finger/wrist movements	United States	[92]
*Type of instrument not specified (* *n* = 2 *)*								
61	ABC test for general movement coordination	NS	NS	NS	NS	NS	The Netherlands	[93]
62	The Drawing Test	NS	NS	1–3 years	NS	To assess arm–hand function	NS	[94]

*Note:* For this table, the latest version of all instruments was used. *Performance-based measures*: These are assessment tools that require a client to actively perform specific tasks whilst the assessor observes and evaluates their performance. These measures assess actual abilities in real‐time or simulated tasks rather than just what they *report* they can do and provide objective data on how a person functions in a given activity or environment. *Proxy measures*: These assessments are completed by someone other than the individual being assessed—typically a caregiver, teacher or parent—who provides information about the person′s abilities, behaviour or participation in daily activities. *Classifications*: Refer to structured systems used to categorise and describe aspects of health, function or disability. They help to standardise how the assessor understands and communicates about clients′ abilities and limitations.

Abbreviations: BED, below elbow deficiency; BPP, brachial plexus palsy; CP, cerebral palsy; DCD, developmental coordination disorder; ed., edition; No., number; NS, not specified (this indicates that no further information was available, irrespective of additional search).

Table 4 summarises the key specifications of the 62 assessment instruments with regard to the name of the instrument, its abbreviation, the latest available version or edition, the age parameters, target population, purpose of the instrument, type of instrument, country of origin and the primary authors of the instrument. Of the 62 instruments identified, the majority (*n* = 46; 74.2%) were performance‐based, with smaller proportions of proxy measures (*n* = 11; 17.5%), classification systems (*n* = 3; 4.8%) and two tools without a clearly stated format. Over one‐third (36.5%) of instruments were designed for children with specific pathologies, such as cerebral palsy (CP) or brachial plexus palsy (BPP), whilst 34.9% targeted mixed populations and 27.4% did not specify.

All classification systems and most proxy measures were developed for pathology‐specific populations, whereas performance‐based tools were more evenly distributed across population types. These patterns highlight both the predominance of performance‐based tools and the practical reality that, in many contexts, especially in LMICs, non‐hand‐specific, multidomain or pathology‐specific tools may be used to assess the hand function of preschoolers.

Table 5 illustrates the types of assessment instruments with their intended target population. Amongst the 62 assessment instruments reviewed, the majority (*n* = 46; 74.2%) were performance‐based, followed by proxy measures (*n* = 11; 17.5%), classifications (*n* = 3; 4.8%) and not specified (*n* = 2; 3.2%). All the classification instruments (*n* = 3; 4.8%) and most proxy measures (*n* = 7; 11.3%) were designed for children with pathologies such as CP, BPP, congenital below elbow deficiency (BED), visual impairment and juvenile idiopathic arthritis (JIA). In total, 23 (36.5%) instruments were specifically designed for children with pathologies, 22 (34.9%) for children typically or atypically developing and 17 (27.4%) were not stated.

**Table 5:** Type of assessment instruments (*N* = 62) and their intended target population.

Type of assessment	*n*(%)	Target population	*n*(%)
Not stated	2 (3.2)	Not stated	2 (3.2)
Classifications	3 (4.8)	Children with CP or neonatal BPP	3 (4.8)
Proxy	11 (17.7)	Not stated	3 (4.8)
		Children without/with developmental delays or conditions	1 (1.6)
		Children with pathologies such as CP, BPP and JIA	7 (11.3)
Performance‐based	46 (74.2)	Typically developing children	6 (9.7)
		Children with pathologies such as CP, BPP and JIA	13 (21.0)
		Not stated	12 (19.4)
		Children without/with developmental delays	15 (24.2)

Abbreviations: BPP, brachial plexus palsy; CP, cerebral palsy; JIA, juvenile idiopathic arthritis.

In addition to mapping the key specifications of the published hand function assessment instruments, this review included a focused analysis of performance‐based instruments to identify the specific hand function domains assessed and the corresponding assessment items used. Table 6 presents 40 performance‐based instruments grouped into five categories for ease of reference: pegboard or box–block instruments (*n* = 6; 15.0%), global developmental assessments *(* *n* = 6; 15.0%), IHM assessments (*n* = 9; 22.5%), assessments for children with specific pathologies and disabilities (*n* = 13; 32.5%) and other hand function assessments (*n* = 5; 12.5%). This in‐depth synthesis was deemed essential, as the review constituted the first phase of a larger project aimed at adapting or developing a contextually appropriate performance‐based instrument for use in LMICs. The analysis of existing instruments informed the next step to generate a preliminary item pool by drawing on current measures and/or designing new items.

**Table 6:** The performance‐based assessment instruments (*n* = 40) with reference to their hand function (HF) domain/s and a brief description of their hand function assessment items.

No.	Name of instrument	HF domain/s included in the instrument	Brief description of the hand function items for the 2–6‐year age range	Quick link (DOI or website)
*Pegboards or box/block tests (* *n* = 7 *)*				
1	Annett Pegboard or Peg Moving Task (PEGS, PMT and PMT‐5)	Grasp and release, hand preference and motor speed	Each of the five pegs is moved one by one to the hole in the opposite row as fast as possible.	https://www.sciencedirect.com/science/article/pii/S0010945213802298
2	Box and Block Test (BBT)	Unilateral manual dexterity and motor speed	Move the maximum number of blocks one by one from one compartment of a box to another of equal size within 60 s.	https://www.physio-pedia.com/Box_and_Block_Test
3	Computerised Peg Moving Task (CPMT) (Annett PMT)	Hand kinematics	Using the Annett Pegboard with an infrared motion‐tracking system.	10.3758/BRM.40.2.503
4	Functional Dexterity Test (FDT)	Grasp and release, tripod pinch, in‐hand manipulation and motor speed	All the pegs are turned over as quickly as possible in the specified order by manipulating each peg in the hand (16 cylindrical pegs arranged in four rows of four pegs each).	10.1097/BPB.0000000000000719
5	Grooved Pegboard	Fine motor dexterity, visual–motor coordination and motor speed	Pegs with a key along one side must be rotated to match the hole before they can be inserted, requiring visual–motor coordination. Performed with dominant and nondominant hands.	https://lafayetteevaluation.com/products/grooved-pegboard
6	Nine‐Hole Peg Test (NHPT)	Finger dexterity, not divided into subdomains	Take the pegs from a container, one by one, and place them into the holes on the board as quickly as possible using only the hand being evaluated.	https://www.physio-pedia.com/Nine-Hole_Peg_Test
7	Purdue Pegboard (PPT)	Gross movement of the fingers, hands and arms; fingertip dexterity and motor speed	The total number of pins placed in the right, left and both hand/s column using the hand/s in the allotted time. The total number of pins, washers and collars assembled in the given time.	https://lafayetteevaluation.com/products/purdue-pegboard
*Global developmental assessment instruments (* *n* = 6 *)*				
8	Bayley Scales of Infant and Toddler Development (Bayley‐4)	Reach, grasp and bilateral hand movement	24–42 months: Threading beads, drawing vertical lines and completing puzzles.	https://www.pearsonclinical.co.uk/store/ukassessments/en/c/Bayley-Scales-of-Infant-and-Toddler-Development-%257C-Fourth-Edition/p/P100040003.html
9	Bruininks‐Oseretsky Test of Motor Proficiency (BOT‐3)	Fine motor precision, fine motor integration, manual dexterity and upper limb coordination	Fine motor precision: Writing, folding and cutting. Fine motor integration: Copying shapes/cubes. Manual dexterity: Making crossed lines in circles, drawing arcs and transferring tokens. Upper limb coordination: Rolling a ball.	https://www.youtube.com/watch?v=e6jOlooa_50
10	Early Childhood Developmental Criteria (ECDC)	Fine motor coordination, not divided into subdomains	Stringing beads, putting small pellets in a container, pencil/crayon drawing tasks and cutting tasks.	10.1177/008124630003000304
11	Malawi Developmental Assessment Tool (MDAT)	Fine motor skills, not divided into subdomains but into activities	34 activities such as reaching and grasping large objects, transferring objects between hands, picking up seeds with a pincer grip, building a tower with blocks, scribbling and copying shapes on paper, putting pegs in a board in 30 s, unscrewing and screwing a bottle cap, picking up sticks and making objects with clay.	https://www.globalhealthlearning.org/sites/default/files/page-files/Malawi%2520development%2520assessment%2520tool_1.pdf
12	Movement Assessment Battery for Children (MABC‐3)	Manual dexterity, not divided into subdomains	Drawing circles, posting coins (preferred and other hand), threading beads and threading lace.	https://www.pearsonassessments.com/content/dam/school/global/clinical/us/assets/mabc-3/mabc3-scoring-report-update.pdf
13	Peabody Developmental Motor Scale (PDMS‐3)	Fine motor section: Hand manipulation subtest and eye–hand coordination subtest	Grasp objects, stack blocks, trace/draw figures/connecting dots, imitate finger–thumb pattern, cutting of paper, stringing cubes in 40 s and manipulate objects.	https://www.pearsonassessments.com/content/dam/school/global/clinical/us/assets/pdms/pdms-3-detailed-narrative-report.pdf
*In-hand manipulation assessment instruments (* *n* = 9 *)*				
14	In‐hand Manipulation Test Quality section (IMT‐Q)	Translation finger–palm and palm–finger, complex rotation and shift, simple rotation and shift and stabilisation	Picking up coins and placing them in a bank, picking up chips and placing them in a container, removing and replacing small bottle lids, picking up and placing cubes, turning over cubes, picking up, turning over and placing pegs, picking up writing tools, putting a key into a lock, turning pages in a magazine and picking up cards.	10.5014/ajot.47.6.505
15	Modified In‐Hand Manipulation Test (TIHM‐M)	Translation finger–palm and palm–finger, complex shift and simple and complex rotation	Using the nine‐hole pegboard and a stick for rotation of five pegs, translation of three pegs, translation of four pegs, translation of five pegs and shift with a stick.	10.32598/irj.17.3.279
16	Observation Protocol: In‐Hand Manipulation and Functional Skill Development	Translation finger–palm, translation palm–finger and complex rotation	Feed the Cookie Monster with objects like beads; flipping a marker so that the tip can write, flipping a peg upside down (pretending it is a candle) and placing it on a cake‐style pegboard; functional activities (pencil rip, holding spoon, buttoning and putting peg in bag).	10.1080/J006v13n03_06
17	Timed Test of In‐Hand Manipulation (T‐TIHM)	Translation finger–palm, translation palm–finger and complex rotation	Picking up two, three, four and five pegs from the pegboard to the hand; manipulating them and placing them back in the pegboard; rotating some of the pegs 360°	10.1002/oti.1385
18	Test of In‐Hand Manipulation (TIHM)	Translation finger–palm and palm–finger and complex rotation	Using the nine‐hole pegboard for translation (finger–palm and palm–finger) and complex rotation of pegs	10.5014/ajot.50.1.52
19	Test of In‐Hand Manipulation—Revised (TIHM‐R)	Translation finger–palm and palm–finger and complex rotation	Rotation (five pegs), translation (four pegs) and translation (five pegs)	10.1177/0308022615600179
20	Test of In‐Hand Manipulation Skills (TIMS)	Translation finger–palm, translation palm–finger, simple and complex rotation and stabilisation	Pen/pencil activity; coins, cubes, beans and jar activity; clay activity. Pegs shaped like a man and a pegboard, keys and lock activity. Playing cards, story book, plastic top, nut and bolt, paper, colour and paper clip activity.	10.4103/2278-344X.194092
21	Unnamed in‐hand manipulation test of Pehoski et al.	Translation finger–palm, translation palm–finger and complex rotation	Using a unique 10‐hole pegboard: Pick up two to five pegs, one at a time; store them in the hand; place them in the pegboard.	10.5014/ajot.51.7.544
22	University of the Free State In‐Hand Manipulation Checklist (UFS IHM‐C)	Translation finger–palm and palm–finger, complex and simple rotation, simple shift and rotation and stabilisation	Using the nine‐hole pegboard and a piggy bank for tasks: Picking up dowels, holding them within the palm and placing them in the pegboard one at a time. Picking up two coins and placing them in the piggy bank slot. Holding a coin horizontally and rotating it with the fingertips.	10.17159/2310-3833/2016/v46n2a9
*Instruments designed for children with pathologies/disabilities (* *n* = 13 *)*				
23	Assisting Hand Assessment (AHA)	Bimanual hand use	Young children up to 5 years play exploratively with the toys in the test kit. For older children (6–12 years), a specially designed board game in two different versions (alien game and fortress game) is used.	https://cpteaching.com/aha-assessments/aha-18-18/?v=959848ca10cc
24	Besta Scale	Grasp, spontaneous use and activities of daily living	Palmar grasp with a cube, pincer grasp with a marble and structured activities requiring both hands using equipment standardised by age (e.g., a 3‐year‐old tears a piece of paper).	https://www.researchgate.net/publication/261734893
25	Both Hands Assessment (BoHA)	Bimanual hand use and hand use quality difference between the affected and unaffected hand	Observation of bimanual play using the same activities as used in the AHA. Thus, children up to 5 years play freely with the toys in the AHA test kit, and older children play board games.	10.1080/01942638.2017.1318431
26	Brachial Plexus Outcome Measure (BPOM)	Wrist, finger and thumb scale (bilateral and unilateral)	Opens a large container, pulls apart the theraputty and strings a bead.	10.1016/j.jht.2012.05.002
27	Computer game–based upper extremity assessment of manual dexterity (CUE)	Object manipulation (unilateral one hand at a time)	Five object manipulation tasks using a peanut ball, soccer ball, tethered tennis ball, cone and plastic ring with a wireless inertial mouse (IB mouse).	10.1177/20556683211014023
28	Erhardt Developmental Prehension Assessment (EDPA)	Grasp and release and object manipulation	Grasp and release of cubes, cylinders/dowels pellets and buttons.	10.1177/000841749105800205
29	Melbourne Assessment of Unilateral Upper Limb Function (MAUUF)	Accuracy of reach and placement, dexterity of grasp and release, object manipulation and fluency of movement	Reach forwards and sideways, grasp, release and draw with a crayon, drawing grasp, grasp and release of a pellet and manipulation of objects.	https://www.rch.org.au/melbourneassessment/
30	Paediatric Arm Function Test (PAFT)	Unilateral arm use and bilateral arm use	Tasks 1–17 unilateral: Reach above head for a ball, reach at waist level for a ball, reach across midline at chest level for ball, grasp of small ball, carry ball, release ball into cup, pour ball out of cup, throw ball onto target, wave bye‐bye, protective extension on the involved side, isolated index finger use (keyboard), grasp of large knob puzzle piece, crayon grasp, crayon use, grasp cracker‐sized food item, grasp small food item (cheerio) and eat with a spoon. Tasks 18–26, both hands: Separate pull‐apart toy, carry large ball, throw large ball through hoop, place hat on head, put on boots and quadruped weightbearing and crawling.	https://www.uab.edu/citherapy/images/pdf_files/CIT_PAFT_Manual.pdf?utm_source=chatgpt.com
31	PizzaPutty Test	Grip function	It uses theraputty, a variety of bead sizes, and a set of instructions to guide patients through a series of activities leading to the creation of a simulated pizza, whilst assessing seven different grip functions.	10.1002/art.38508
32	Shriners Hospital for Children Upper Extremity Evaluation (SHUEE)	Spontaneous functional analysis for five upper extremity limb segments (thumb, fingers, wrist, forearm and elbow)	The first four tasks related to the thumb and finger: Money from the wallet and string beads. (The rest of the task requires wrist, forearm and elbow movement).	https://cdn-links.lww.com/permalink/jbjs/c/jbjs_2017_03_12_davids_326_sdc1.pdf
33	Test for Manual Dexterity in Visually Impaired Children	One‐handed skills, two‐handed skills, perception of movement and hand–eye coordination	Eight motometric items and motoscopic observation (qualitative aspect of movements). Items 1 and 2 test one‐handed skills (putting coins in a piggy bank and putting rings on pegs). Items 3, 4 and 5 test two‐handed skills (screwing two nuts on a bolt, threading beads and threading a cord through a board). Items 6 and 7 test the perception of movement (kinesthetic acuity test and kinesthetic memory trace). Item 8 (only for children with low vision) tested hand–eye coordination (placing dots).	10.1177/0145482X9909301003
34	Unilateral Below Elbow Test (UBET)	Prosthesis‐on domain and prosthesis‐off domain	Different tasks for age groups. Age 2–4: Take Play‐Doh out of a plastic baggie, bang cymbals together, put a sock on a foot, thread beads, open a jar of bubbles, open a bag with Duplos, open a new box of eight crayons and remove one. Age 5–7: Cut a paper circle, remove the cap from the felt tip marker, sharpen a pencil, do buttons on a vest, tie shoelaces into a knot, turn the kaleidoscope and separate Lego blocks.	https://wendytomhave.com/ubet
35	Quality of Upper Extremity function (QUEST)	Grasp	Items related to grasp: Independent thumb and finger movement, grasp of a cube, grasp of cereal and grasp of pencil/crayon.	https://canchild.ca/en/shop/19-quality-of-upper-extremity-skills-test-quest
*Other hand function assessments (* *n* = 5 *)*				
36	Assessment of Children′s Hand Skills (ACHS)	Manual gesture, body contact hand skills, grasping, holding, in‐hand manipulating, releasing, isolated finger movement, reaching, turning, carrying, moving, catching, throwing and stabilising, transferring, using both hands simultaneously and using both hands cooperatively	20 hand skill items grouped in four domains. Leisure and play domain: Block, puzzle, stringing beads, catching and throwing objects, card game, play dough, folding paper and handling money. School/education domain: Turn book, drawing or colouring, writing and copying, cutting paper, pasting, using a computer, using school tools (ruler) and putting on a backpack. Activities of daily living domain: Drinking, eating, dressing upper body, putting on socks and shoes, washing hands and brushing teeth.	10.5014/ajot.2010.08158
37	Jebsen‐Taylor Hand Function Test (JTHFT)	Fine motor movements and hand function activities (weighted and nonweighted)	Writing, card turning, picking up small common objects and placing them in a container, stacking checkers, simulated feeding, moving light objects and moving heavy objects.	10.5958/0973-5674.2017.00092.2
38	Strength–Dexterity Test (SDT)	Pinch force (strength) and directional accuracy (precision)	Based on the ability to use pinch to fully compress springs with different requirements of strength.	https://valerolab.org/Papers/Valero2002Strength.pdf
39	Task‐Based Bilateral Fine Motor Skill Assessment (TBA)	School readiness task‐based fine motor activities	Tasks include writing, drawing around an object, tearing on a line, threading beads, tying shoelaces, cutting around a circle and a square and buttoning a shirt.	https://scielo.org.za/pdf/sajot/v43n1/03.pdf
40	The Bead Maze Hand Function Test for Children (BMHF)	Force control of fingers, task completion and time	Fingers move a bead over wires (toy enhanced with force sensors).	https://research.aota.org/ajot/article/78/4/7804205010/25218/The-Bead-Maze-Hand-Function-Test-for-Children

*Note:* The assessment item refers to the movement, task and/or activity performed or asked about in the instrument. No abbreviation of the instrument is listed if it was not identified in the literature. The latest edition of all instruments was used. Only the hand function domain included in the instrument (with the terminology used in the instrument′s publication) is presented in this table, and domains unrelated to hand function are not presented in the table. Some of the performance‐based tests listed in this table (such as the BOT‐2, PDMS‐2 and MABC‐2) also include proxy‐report measures or brief observational checklists as part of their assessment kits. If no further information about an instrument was available about the hand function domains and items (through additional websites, library assistance and author/publishing company correspondence), or no English translation was available, the instruments were excluded from this table. On further investigation, it was found that some of the details provided in the publications used to compile Table 4 were incorrect. The study′s eligibility criteria were therefore reapplied, resulting in the exclusion of the following assessments (cf. Table 4): Kobe Test, Developmental Hand Function Test, Brazilian Scale for Motor Development (MDS), Motor Performance Checklist (MPC), and Toddler and Infant Motor Evaluation (TIME).

Abbreviation: DOI, digital object identifier.

## 4. Discussion

This scoping review mapped published instruments for assessing hand function in preschoolers, with in‐depth analysis of performance‐based tools due to their particular relevance to occupational therapy. Proxy‐report measures, classification systems and global developmental assessments with discrete fine motor or hand function subscales were also included, as such tools are often the only feasible option in low‐ to middle‐income settings. Instruments designed for specific clinical populations were eligible if they assessed skills relevant to typically developing children, and tools with broader age ranges were included when the 3–5‐year band fell clearly within scope. This inclusive mapping reflects real‐world assessment practice, whilst the focused analysis of performance‐based tools identifies domain coverage and item gaps to inform future tool adaptation or development.

### 4.1. Demographic Information

The demographic findings indicated an increase in publications from 2010 onwards, which could possibly be attributed to the growing interest in early diagnosis and treatment of developmental delays and disorders affecting hand function in children ([14, 1, 17]). Furthermore, the emphasis on evidence‐based practice has increased the demand for high‐quality assessment tools in clinical and research healthcare settings, resulting in more sources reporting on instrument development, standardisation and cross‐cultural adaptations of instruments [9, 95, 96].

The limited number of publications detailing systematic instrument development and the psychometric properties of tools was pointed out. This may partly account for the gaps identified during the first round of analysis related to key specifications, domains and items that necessitated a secondary data extraction method (Figure 1). However, in some cases, it is possible that instruments were developed specifically for a particular research study, as noted, although not always explicitly stated, and were never refined further or published. In other cases, intellectual property restrictions may limit access to full details, such as items and tasks. Despite these challenges, extensive literature is available to guide systematic instrument development [97–100] and how to critically appraise instruments [101].

Congruent with the purpose of a scoping review, this study allowed for the inclusion of multiple methodologies, to enable a comprehensive overview of the existing literature ([102, 103]). Most included studies (Table 2) focused on intervention designs, likely reflecting a growing interest in evidence‐based approaches to improving hand function [96, 104]. This was followed by descriptive studies, which may indicate efforts to understand current practices or populations, and psychometric research, highlighting the ongoing need to validate and refine assessment instruments [18]. Many of the included studies referred to multiple assessment tools, which may reflect the complexity of hand function and the absence of a universally accepted gold standard instrument and underscore the importance of developing a comprehensive, standardised assessment tool that covers all domains of hand function.

### 4.2. Key Specifications of the Assessment Instruments

To the authors′ knowledge, this scoping review is the first to report on all types of hand function instruments (Table 4) for preschool children, providing a broad overview of their key specifications as detailed in the sections that follow. The wide variety of available assessments reflects the diverse needs of paediatric occupational therapy.

Most of the instruments lacked clear information about their *edition/version*, and those that did provide such details were often not in their first revision, suggesting ongoing refinement, adaptation and validation over time. This might highlight the iterative timeous process of developing assessment instruments and the importance of refinement in response to research, contextual feedback or technological advances [97, 99, 105].

Hand function assessment instruments vary widely in *age parameters*, with some targeting narrow developmental stages and others covering broad age ranges. However, instruments with wide age parameters may lack the sensitivity to detect subtle, age‐specific motor milestones, particularly during early childhood when development is rapid and nonlinear [7, 106]. Selecting an age‐appropriate instrument is therefore critical to accurately assess developmental expectations and functional motor performance, ensuring that assessments align with the child′s specific stage of growth ([4, 107]).

As shown in Table 5, many reviewed instruments were developed for children with specific pathologies or developmental delays, whilst fewer targeted typically developing children, and several did not specify a *target population*. This likely reflects the clinical need for tools that support identification, intervention and funding for children with known impairments [25]. Moreover, children with visible functional limitations are more frequently referred for assessment, prompting the development of condition‐specific instruments [39]. The predominance of pathology‐specific instruments and the scarcity of tools for typically developing children highlight a gap in the availability of performance‐based assessments suited for universal preschool use. Furthermore, unclear target population specification may limit generalisability and appropriate use of instruments, particularly in early intervention or research contexts. Clear reporting on target populations is essential to ensure appropriate tool use and interpretation [105].

A broad range of *instrument purposes* was identified. Most were designed to assess (therapist‐directed) specific aspects of hand function and other developmental components. Others aimed to capture the perceptions and concerns of parents or the child (proxy‐reported), whilst some focused on classifying children by severity or functional levels [7]. This variety reflects the evolving understanding that hand function cannot be fully captured by a single method or perspective. Combining direct assessments with proxy input from parents or children offers a more holistic and real‐world view of a child′s hand function in everyday contexts. Such a collaborative approach can better inform intervention planning and support family‐centred care [108].

Mainly three *types of instruments* aimed at assessing hand function were identified, with the majority performance‐based, followed by proxy measures and classifications, all equally important depending on the purpose of the evaluation. The use of proxy measures became more widely used during the shift towards family‐centred care, supported by evolving healthcare models recognising the role of family and the environment in child development [109, 110]. Performance‐based instruments have their advantages, since they enable therapists to directly observe task performance, reducing reliance on subjective recall [111]. However, a closer examination of performance‐based tools shows that most were developed for clinical populations, focusing on impairments rather than functional performance in typically developing preschool children′s everyday occupations. This imbalance means that occupational therapists, particularly in LMICs, may need to adapt pathology‐specific measures or rely on global developmental tools, which may not fully capture typical preschool hand function.

### 4.3. Country of Origin

The geographic origin of an assessment instrument can influence its applicability across contexts. Most of the identified instruments were developed and used in high‐income, Global North settings, with fewer than one‐third originating from or being used in LMICs or Global South contexts. This imbalance may introduce global and socioeconomic bias, as many tools lack cultural and linguistic neutrality, having been designed primarily for English‐speaking, urban and relatively affluent communities. Moreover, many assessment items depend on culturally specific activities; materials, such as branded toys or local currency (e.g., specific coins) and developmental milestones, which may not be relevant for LMICs.

From an occupational therapy perspective, this matters because hand function assessments need to reflect children′s real‐world occupations and contexts. Although some studies have reported on the translation and adaptation of instruments, limited evidence is available on the methods used or the extent to which these adaptations account for social, cultural and contextual relevance [112]. Yet, societal and cultural contexts profoundly shape meaning, values, habits and interaction with objects—factors that directly influence hand use and performance [95, 96]. Therefore, careful consideration of these elements is essential when adapting, translating or renorming an instrument to ensure its relevance and validity within LMIC contexts.

### 4.4. Performance‐Based Instruments

Several discussion points regarding the domains, items and type of instrument emerged when mapping out the performance‐based instruments (Table 6).

#### 4.4.1. Coverage of Hand Function Domains

At face value, many instruments appear to assess the full repertoire of hand function. For example, an instrument may claim to evaluate “dexterity,” but upon closer analysis, it becomes evident that it only measures limited components, such as grasp and release. This discrepancy may stem from a key challenge identified in the review—the inconsistent use of terminology across instruments.

Terms such as fine motor dexterity, manual dexterity and fine motor coordination are used interchangeably, yet it remains unclear which specific hand function domains are being assessed. This inconsistency, when considered against the broad definition of hand function, hampers comparability between instruments, as it is unclear whether similarly named domains truly assess the same construct.

Furthermore, when considering all the hand function domains according to the existing *children′s hand skills framework* [3] and the *functional repertoire of the hand* model [5], a clear lack of representation of certain hand function domains is evident. The domain where the hands are not involved whilst in contact with objects (i.e., *manual gestures* and *body contact*) was only represented in one assessment [3]. *Manual gesturing* is an important complementary communication tool that allows children to express feelings, attitudes and ideas by means of, for example, waving, signing and clapping. The *body contact* hand skills, such as scratching, rubbing and touching, enable children to use their hands to support functional self‐care needs [3].

Several instruments included domains of grasp, hold and release of pegs, blocks and other objects. The domain of IHM was well covered, although not all instruments encompassed all the IHM components. However, other aspects of object manipulation as described by Kimmerle et al. [5], such as pushing, rotating, orienting, guiding, inserting and removing, are not explicitly listed as domains. Two instruments include the domain of isolated finger movements (the Paediatric Arm Function Test [PAFT] and the Assessment of Children′s Hand Skills [ACHS]; see Table 6). Regarding the bilateral hand use domain, some instruments have elements of bilateral hand function, mostly regarding using both hands cooperatively, for example, stringing beads. However, only one test explicitly refers to the terms *simultaneous* or *cooperative* bilateral movements [34].

Hand function is a complex performance skill that depends on the dynamic interplay of multiple components [7]. However, the review revealed that many instruments primarily target isolated client factors, such as body functions. For instance, the Box and Block Test (BBT) focuses on motor speed and dexterity. In contrast, an instrument such as the ACHS takes a more holistic approach guided by considering the interconnectedness of the child′s occupations, context, performance patterns and skills [104, 113]. This range of instrument types ensures that the mapping reflects the diversity of measures currently in use, many of which may contribute to relevant domains or items even if their primary aim is not occupational performance.

#### 4.4.2. Hand Function Items

In this study, the *items* refer to the specific movements, tasks or activities that a child is asked to perform, along with the associated objects, apparatus or equipment used. These tasks are designed to assess various components of hand function in preschool children.

Tasks commonly include play‐based activities such as stringing beads or cubes, screwing and unscrewing containers, manipulating coins and piggy banks, handling play dough, solving puzzles and playing board games. Preschool‐related, paper‐based tasks such as folding, drawing, cutting, tearing and line tracing are also included frequently, reflecting school readiness and early academic fine motor demands. Additionally, activities of daily living (ADLs) such as simulated feeding with a spoon, buttoning a shirt, tying shoelaces or putting on shoes are incorporated into several instruments to assess hand function.

A number of instruments make use of nonfunctional (abstract) object manipulation, such as inserting pegs, pins, blocks or pellets into pegboards, containers or boxes. Whilst these tasks are often unfamiliar to the child, they offer standardised and simple formats for assessing specific fine motor components [7, 107]. Some instruments include more unfamiliar items for children in LMICs, such as using a computer mouse, manipulating Legos or turning a kaleidoscope. Of interest is that most instruments tend to employ simple, familiar and culturally/gender‐neutral objects, ensuring broader applicability across diverse settings. This raises important considerations about the cultural appropriateness and accessibility of hand function assessment instruments [114].

It is evident in some instruments that an age‐appropriate difficulty progression is followed and clearly indicated in the item instructions. The items are either staged by increasing complexity (e.g., stacking fewer blocks at 3 years vs. more blocks at 5 years) or through the use of different tasks (e.g., younger children stack blocks, whilst older children build puzzles). However, some instruments use the same items across all age groups, with grading based on the number of pegs manipulated or the speed and accuracy of task performance, as reflected in the scoring model. The challenge arises when instruments with similar items for all ages lack clear age indicators in the scoring model, making it difficult to pinpoint exactly where a child′s ability lies, as indicated in some of the IHM assessments.

Most hand function skills in preschool children are assessed through interactions with objects, with the exception of tasks involving manual gestures. The properties of these objects—such as size, shape, weight, texture, spatial orientation and quantity—play a critical role in determining how a child engages with and performs hand function tasks [107]. For example, screwing on a medium‐sized lid with grooves for better grip may be easier for a child than turning a small, smooth‐edged coin due to differences in texture, size and required precision.

The item‐to‐domain mapping appears relevant across instruments, with tasks corresponding well to the intended domain. For example, a task such as picking up blocks and placing them into a box appropriately targets grasp and release skills, which are foundational components of hand function. There is, however, notable variation in the comprehensiveness of item pools. Some instruments offer a comprehensive variety of items that thoroughly cover a specific domain. For instance, the Bead Maze Hand Function (BMHF) test includes a range of items designed to comprehensively assess bilateral fine motor coordination within one domain. Conversely, other instruments may aim to assess multiple domains, such as the components of IHM, but include only a single item (e.g., a pegboard), raising concerns about content validity and measurement sensitivity [5, 48].

The combined item pool mapped from all the performance‐based instruments provides valuable insight into the tasks currently used to assess hand function in preschool children. By analysing the nature of these items, researchers can identify strengths and limitations across instruments. This synthesis may serve as a practical guide for adapting existing tools or informing the development of more comprehensive, contextually appropriate, and developmentally sensitive assessment instruments for young children.

### 4.5. Types of Performance‐Based Instruments

Closer examination of *pegboard/block instruments*, such as the Box and Blocks and Functional Dexterity Test (FDT), reveals they were initially developed for adults and later adapted for children, whilst some were specifically designed for paediatric use. Few instruments, such as the Grooved and Purdue pegboard tests, can be used across an individual′s lifespan. These tests are widely used by various professionals other than occupational therapists and have been well researched. Furthermore, these tests have the advantage of short administration time and simple procedures and require minimal equipment. However, it mostly covers unilateral object‐related movements and should be complemented with other items to cover all hand function domains. Notably, motor speed was one of the most frequently included domains, contrary to only a few other assessment instruments that incorporated it. The speed or pace, rhythm and sequence of a movement form an important measurable quality aspect of hand function that can be used to compare baseline function with performance following an intervention [106, 107].

Investigating the *global developmental assessments* revealed that their value lies in the ability to cover multiple domains of overall developmental functioning and to identify children requiring more domain‐specific assessment. However, for a therapist requiring a detailed hand function assessment, some instruments may lack a comprehensive representation of all hand function components. Most of these instruments have undergone revisions, contain well‐structured manuals and are widely used in both clinical practice and research. These instruments′ item sets have clear assessment and scoring guidelines for age intervals to guide the adaptation or development of a preschool‐specific hand function assessment [56].

The *IHM tests* were most specific to what aspect of IHM is assessed, as well as what the assessment items comprise. These items would be useful to consider in an item pool for further adaptation/development of a hand function instrument. However, as noted by Kruger et al. [24], most instruments are not commercially available for purchase but can be accessed upon request.

Several *instruments designed for children with pathologies or disabilities* focused on unilateral or bilateral grasp and release of objects in tasks to assess differences in affected/nonaffected hands. None of the instruments covered all the domains of hand function but consisted of creative play‐based items.

The *other hand function assessments* listed in Table 6 did not fit into a specific classification type. Amongst them, the ACHS stands out as the most comprehensive instrument, covering a broad range of hand function domains through 20 items organised according to occupational areas (e.g., play and school). It is also the only instrument grounded in a theoretical framework [3, 34].

Notably, the Jebsen‐Taylor Hand Function Test (JTHFT) is the only instrument that incorporates object properties related to weight and includes both weighted and nonweighted items. According to Henderson and Pehoski [107], the weight of an object influences anticipatory control and the force with which a child manipulates the object.

The BMHF Test for Children makes a unique contribution to traditional hand function assessments by incorporating elements of dynamic motor movement and force regulation in a continuous, real‐time task, enhanced by an electronic component that enables objective measurement and reduces observer bias [73, 73]. This innovation aligns with the broader trend towards digitally integrated assessments, reflecting the growing presence of technology in preschoolers′ daily lives. As tablets, touchscreens and computer‐based activities increasingly shape how fine motor and hand function skills develop, traditional tools may fail to capture emerging digital‐specific skills such as tapping, dragging and stylus control. Given the inconsistent effects of touchscreen use on motor development, there is a pressing need for updated instruments that integrate media‐ and technology‐specific tasks. Tools such as the BMHF and other tablet‐based platforms [10, 115–117] are essential to ensuring hand function assessments remain relevant to the contemporary developmental context in both high‐ and low‐income settings.

In summary, a wide range of hand function assessment instruments is available, each designed for specific purposes and targeting either single or multiple domains of hand function. However, only one instrument—the ACHS—was found to comprehensively cover most of the domains. The mapped item pool revealed a notable gap in media‐ and technology‐specific tasks, underscoring the need for instruments that align with contemporary childhood experiences. Nevertheless, the item pool may offer a foundation from which new or adapted items can be developed. The findings further point to the importance of incorporating occupation‐based, preschool‐relevant tasks using familiar objects and equipment. It is suggested that particular attention should be given to object properties and ensuring age‐appropriate progression in task difficulty to enhance developmental relevance and accuracy in assessment.

### 4.6. Limitations

Several limitations warrant consideration in this study. (i) The term *hand function* is used interchangeably and often synonymously with terms such as hand skills, dexterity, fine motor skills and fine motor coordination. This lack of consistency in terminology complicates the classification of domains. Moreover, hand function is a broad term that may encompass numerous components. Accordingly, the selection criteria (Table 1) were designed to accommodate a broad range of terms related to hand function whilst carefully excluding studies that referred exclusively to individual performance components, such as muscle tone, that do not reflect the broader concept of hand function. (ii) Given the large number of instruments identified, this review mapped key specifications of the reported instruments but did not systematically assess their psychometric properties, clinical application and other practical qualities. The lack of detailed reporting on the assessment instruments used in the included publications resulted in gaps in the mapped evidence and necessitated the inclusion of additional sources. This additional search via other methods (Figure 1) facilitated clarifying the eligibility of certain instruments, leading to the exclusion of those not meeting the selection criteria and the inclusion of one additional instrument. (iii) Incomplete reporting in many publications required supplementary sources to verify eligibility and specifications, which may have introduced bias. (iv) No formal critical appraisal of methodological quality was conducted, consistent with the objectives of a scoping review. However, descriptive characteristics of the included sources, such as study design, sample size and research setting, were extracted and reported to provide context for the mapped evidence. (v) Most instruments were developed and/or validated in HICs, restricting the ability to draw conclusions about cultural or contextual appropriateness in LMICs.

### 4.7. Implications for Research and Practice

Whilst the review captured a diverse range of measures, a gap for a performance‐based hand function assessment instrument for preschool children, culturally adaptable for LMICs, was confirmed. From a research perspective, there is a clear need for (i) constructing a review focusing on the detailed content of the instrument, clinical utility and psychometric properties of selected instruments; (ii) incorporating media‐related electronic and digital‐based activities into hand function evaluations; (iii) developing a standardised glossary to define key terms and commonly used synonyms, promoting conceptual clarity and improving comparability across studies and instruments; (iv) developing a conceptual assessment framework clearly outlining and defining concepts, core domains, item sets and assessment levels for both the assessment and intervention process of preschool children′s hand function and (v) adapting/refining existing instruments or developing a new preschool hand function assessment instrument that is grounded in a sound and comprehensive theoretical framework for hand function assessment.

Practically, occupational therapists and related professionals should select tools with careful consideration of purpose, scope and contextual fit, adapting items where necessary to reflect children′s daily occupations and available resources. In settings where performance‐based tools are unavailable, proxy measures and classification systems may serve as interim solutions, but their limitations in capturing real‐world hand performance should be recognised.

## 5. Conclusion

This scoping review represents the first known published synthesis of hand function instruments for preschool children, with a focused synthesis of performance‐based tools identified from existing literature. A comprehensive summary of 62 instruments assessing hand function, categorised as performance‐based measures, proxy measures and classification systems, was compiled. The performance‐based instruments were further analysed and mapped according to the hand function domains assessed and the items included. This provides evidence on the hand function domains covered by each instrument and offers a pool of potential items for future instrument adaptation or development.

The results indicate that none of the identified instruments was developed to assess all components of hand function in a standardised, performance‐based and culturally adaptable manner for preschool children from LMICs. This review provides foundational evidence to inform clinical practice and highlights the need for future research.

## Author Contributions

All content was reviewed and edited by the authors, who take full responsibility for the content of the publication.

## Funding

No funding was received for this manuscript.

## Ethics Statement

As a desk‐based review study with no involvement of human participants was performed, an ethical waiver was obtained from the Health Sciences Research Ethics Committee (HSREC) of the University of the Free State in accordance with the Faculty of Health Sciences ethics review policy.

## Conflicts of Interest

The authors declare no conflicts of interest.

## Data Availability

The data that support the findings of this study are available upon request from the corresponding author.
